# Supplementary material for: An Alzheimer’s Disease Patient-Derived Olfactory Stem Cell Model Identifies Gene Expression Changes Associated with Cognition
Source: Cells. 2022 Oct 17;11(20):3258. doi: 10.3390/cells11203258 (PMC9601087; doi:10.3390/cells11203258)
Supplement: Supplementary file 1 [file cells-11-03258-s001.zip › Supplementary materials.pdf]

SUPPLEMENTARY MATERIALS

Supplementary Figures

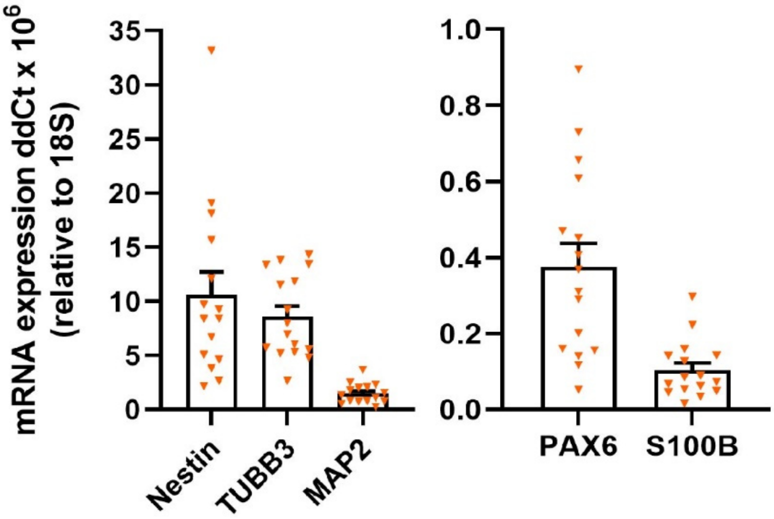

Figure S1. Characterisation of ONS cells by qRT-PCR. Relative expression of mRNA for *Nestin* and *PAX6* (stem cell markers), *TUBB3* and *MAP2* (markers for immature neurons) and *S100B* (glia marker) in HC, MCI and AD ONS cells (HC *n*=5, MCI *n*=5 and AD *n*=6, 3 technical replicates per line). Data are presented as mean ± SEM.

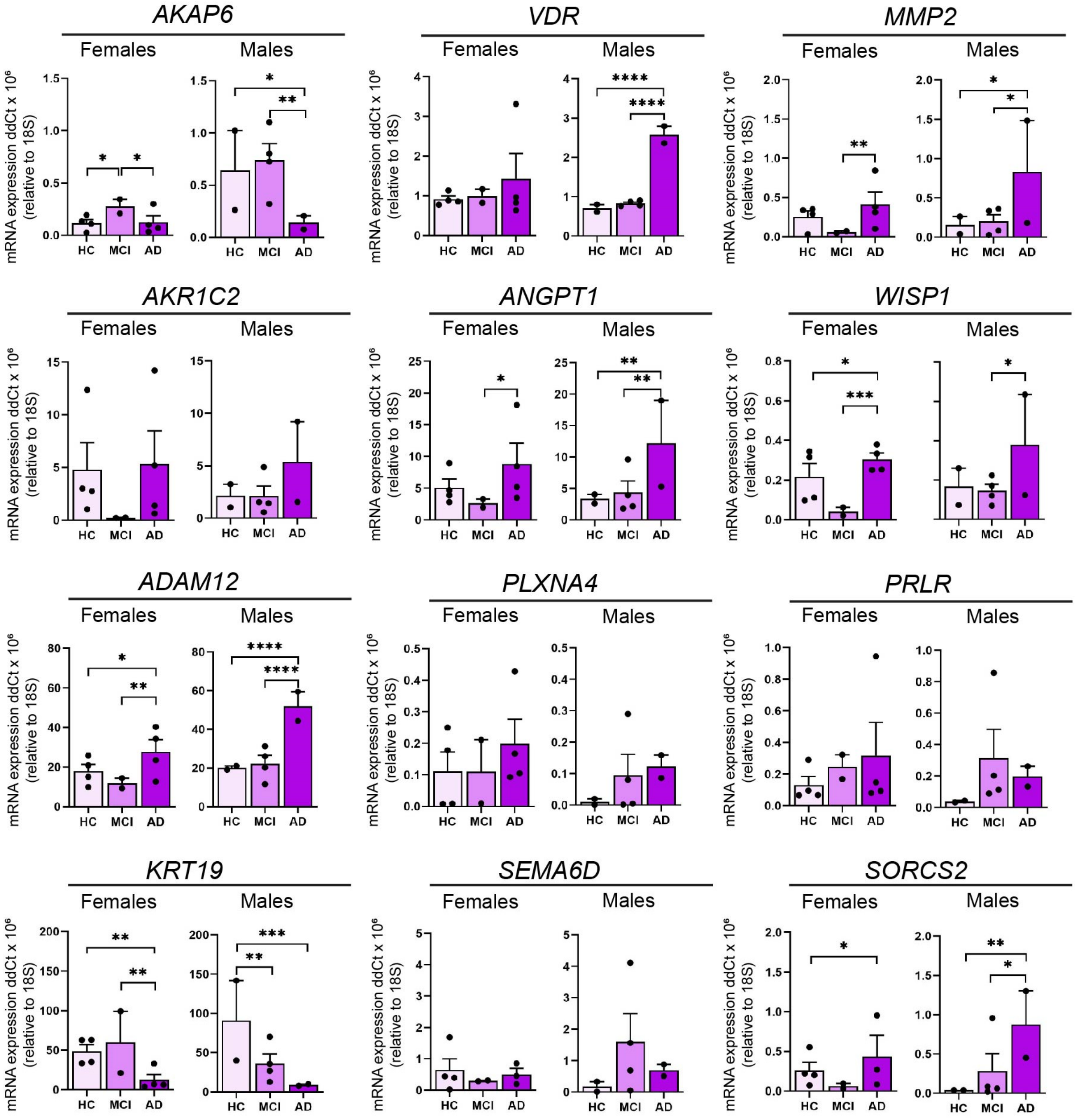

Figure S2. The influence of sex on 12 leading DEGs in ONS cells. Relative expression of mRNA for *AKAP6, VDR, MMP2, AKR1C2, ANGPT1, WISP1, ADAM12, PLXNA4, PRLR, KRT19, SEMA6D* and *SORCS2* in HC, MCI and AD ONS cells (HC females: n=4 lines, HC males: n=2 lines, MCI females: n=2 lines, MCI males: n=4 lines, AD females: n=4 lines, AD males: n=2 lines, in a total of 12 technical replicates from n=4 and 6 technical replicates from n=2). Data are presented as mean ± SEM. Statistical analysis between multiple groups was performed using one-way ANOVA, *$p < 0.05$, ** $p < 0.01$, ***$p < 0.001$, ****$p < 0.0001$.

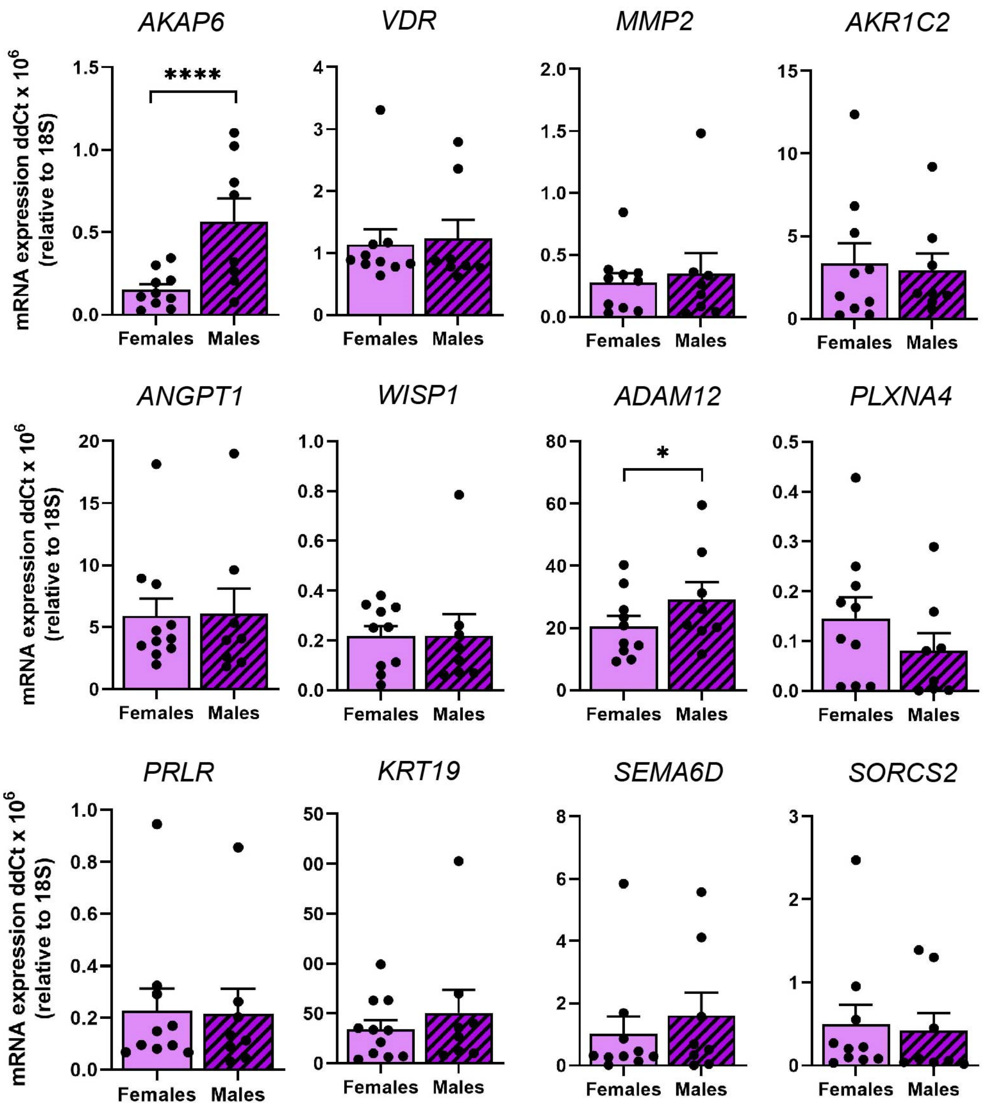

Figure S3. The influence of sex irrespective of clinical status on 12 leading DEGs. Relative expression of mRNA for *AKAP6, VDR, MMP2, AKR1C2, ANGPT1, WISP1, ADAM12, PLXNA4, PRLR, KRT19, SEMA6D* and *SORCS2* in HC, MCI and AD ONS cells (Females: n=10, males: n=8, 3 technical replicates per line). Data are presented as mean ± SEM.

Statistical analysis between multiple groups was performed using one-way ANOVA, *$p < 0.05$,, ****$p < 0.0001$.

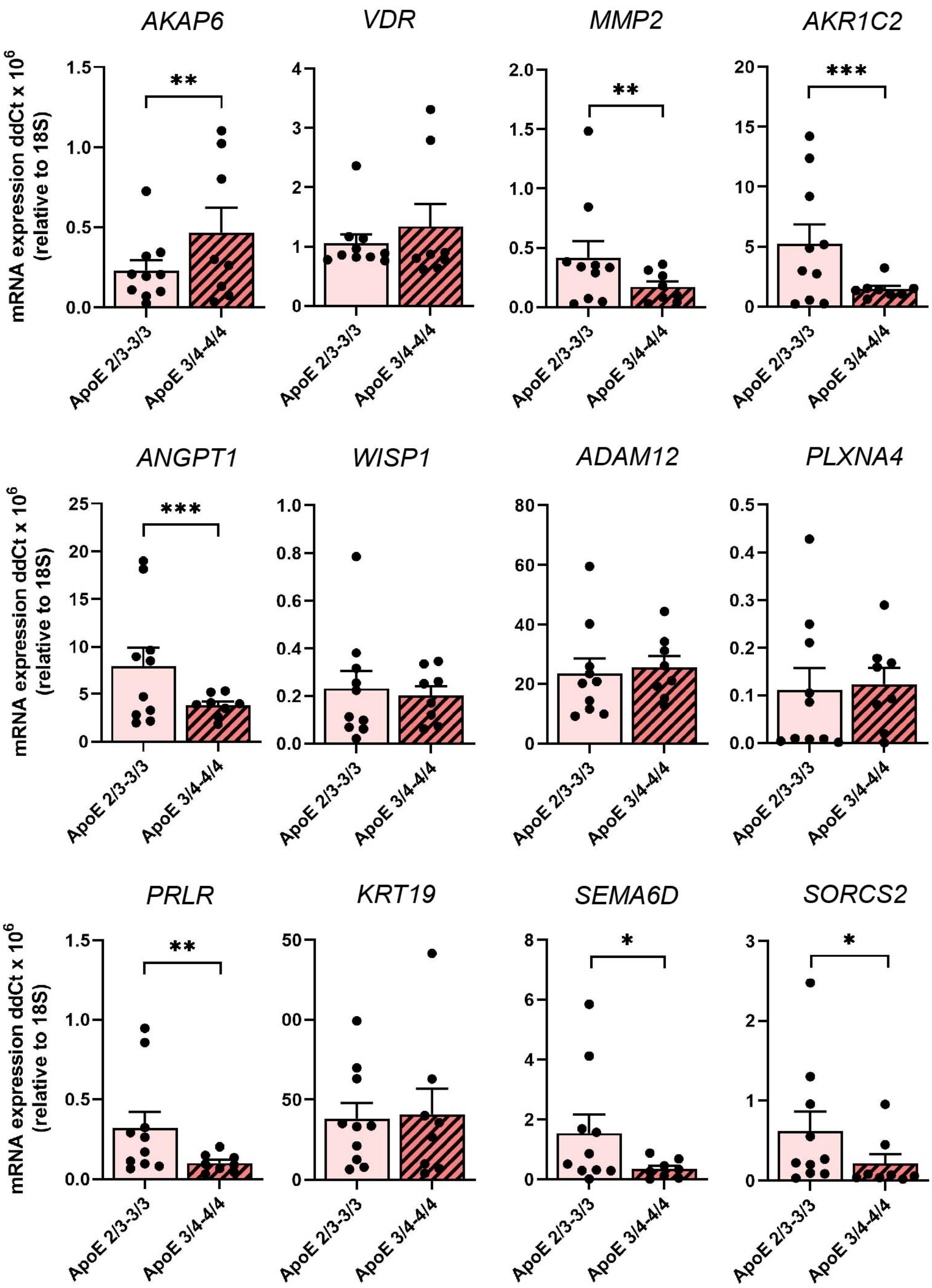

Figure S4. The influence of ApoE genotype irrespective of clinical status on 12 leading DEGs. Relative expression of mRNA for *AKAP6, VDR, MMP2, AKR1C2, ANGPT1, WISP1, ADAM12, PLXNA4, PRLR, KRT19, SEMA6D* and *SORCS2* in HC, MCI and AD ONS cells (*APOE* 2/3-3/3 genotype: n=10, *APOE* 3/4-4/4 genotype: n=8, 3 technical replicates per line). Data are presented as mean ± SEM. Statistical analysis between multiple groups was performed using one-way ANOVA, *$p < 0.05$, ** $p < 0.01$, ***$p < 0.001$

Supplementary Tables

Table S1. Sphere size, morphology, number, and formation time.

Sample ID	Neuro-spheres ready for collection (days)	Neuro-sphere number/ 25 cm^2	Neuro-sphere diameter (µm) (mean ± SD)	Percentage of small neuro-spheres (<60 µm) (%)	Percentage of large neuro-spheres (>150 µm) (%)	Neurosphere morphology
**Healthy Controls (HC)**						
HC1	7	<5	Not available	Not available	Not available	Not available
HC2	6	50-100	129.1 ± 32.2	0	37.5	Compact and round neurospheres.
HC3	5	20-50	129.1 ± 56.5	11.1	44.4	Compact and round neurospheres.
HC4	3	50-100	186.3 ± 102.1	0	60.0	Compact and round neurospheres.
HC5	5	20-50	103.8 ± 38.0	0	12.5	Compact and round neurospheres.
HC6	10	<5	106.7 ± 43.8	0	21.4	Compact and round neurospheres. A few large, asymmetric, and aggregated spheres.
**Avg.**	**6**	**20-50**	**131.0 ±54.5**	**2.2**	**35.2**	
**Mild Cognitive Impairment (MCI)**						
MCI1	12	<5	238.5 ± 82.7	0	100	Compact and round, large-sized neurospheres.
MCI2	7	Not available	Not available	Not available	Not available	Not available
MCI3	7	50-100	122.7 ± 31.8	0	31.3	Most of the spheres are compact and round in shape. Also some larger, abnormal cell clusters.
MCI4	2	50-100	138.5 ± 33.4	0	30.8	Most of the spheres are compact and round in shape.

MCI5	7	50-100	128.1 ± 39.8	0	13.3	Most of the spheres are compact and round in shape. Also some larger, abnormal cell clusters.
MCI6	5	50-100	103.5 ± 31.7	6.3	6.3	Compact and round neurospheres.
**Avg.**	**7**	**50-100**	**146.3 ±43.9**	**1.3**	**36.3**	
**Alzheimer's disease (AD)**						
AD1	5	50-100	113.5 ± 30.2	5.6	0	Compact and round neurospheres with consistent size.
AD2	8	20-50	59.6 ± 15.0	66.7	0	Compact and round neurospheres with very small size.
AD3	7	50-100	142.6 ± 41.1	0	15.4	Compact and round neurospheres. Some large cell aggregates.
AD4	2	50	131.5 ± 81.5	0	15.4	Compact and round neurospheres with mainly consistent size. A few very large spheres.
AD5	10	20-50	102 ± 51.9	0	20	Compact and round neurospheres with mainly consistent size. Some big cell aggregates.
AD6	3	<5	Not available	Not available	Not available	Not available
**Avg.**	**6**	**20-50**	**109.8 ±43.9**	**14.5**	**10.2**	

Table S2. qRT-PCR primer sequences.

Primer	Forward Sequence	Reverse Sequence
AKAP6	TGTGCTCTCAAGGAAGCTGTG	CATGTCCTTCAGTCCTGCTTTG
ANGPT1	TGGCCCAGATACAGCAGAATG	GAAGTTTGATTTAGTACCTGG GTC
MMP2	TCTGTGTTGTCCAGAGGCAAT	TTGATTTGAAGCCAAGCGGTC
VDR	AGACCTCACAGAAGAGCACC	ACGTTCCGGTCAAAGTCTCC
ADAM12	CGCTCGAAATTACACGGGTC	AGTCCCCTGAGACCAGAACA
PRLR	CCACATGAACCCTGAAGTGAA	TGAAAACGGTTGCAGATGCC
SORCS2	TGCTGTTTGTCATCGGGCTC	CCTGGCCGTTTCCTTTTGA
WISP1	AGTGCTGTAAGATGTGCGCT	ACACTTCTTCCCTGCGTGC
KRT19	TGGAGATGCAGATCGAAGGC	CTCAGCGTACTGATTTCCTCCT
AKR1C2	GCCAGGTGAGGAAGTGATCC	CACTTCTCCATGGCCTCCC
PLXNA4	GGCTCCATCTGCAGTGTCAA	AGTCTCCCCTACTGGACGC
SEMA6	GCCCTCTTTGCTGATGGGAA	GCAGATCCATCACCCATGCT
TUBB3	GGCCAAGTTCTGGGAAGTCAT	CTCGAGGCACGTACTTGTGA
NESTIN	CTCAGCTTTCAGGACCCCAA	GTCTCAAGGGTAGCAGGCAA
PAX6	TGGTATTCTCTCCCCCTCCT	TAAGGATGTTGAACGGGCAG
S100B	TTCTGGAAGGGAGGGAGACA	CTCCTGCTCTTTGATTTCCTCT
MAP2	GACTGCAGCTCTGCCTTTAG	AAGTAAATCTTCCTCCACTGTG AC
18S	TTCGAGGCCCTGTAATTGGA	GCAGCAACTTTAATATACGCT ATTGG

Other Supplementary Materials for this manuscript include the following:

Table S3. List of the DEGs with a *p*<0.01
Table S4. List of the DEGs with a *p*<=0.015, log2FC<-0.4 or log2FC>0.4
